# Maternal age effects on offspring lifespan and reproduction vary within a species

**DOI:** 10.1002/ece3.11287

**Published:** 2024-05-16

**Authors:** Alyssa Liguori, Sovannarith Korm, Alex Profetto, Emily Richters, Kristin E. Gribble

**Affiliations:** ^1^ Josephine Bay Paul Center for Comparative Molecular Biology and Evolution, Marine Biological Laboratory Woods Hole Massachusetts USA; ^2^ Department of Biology State University of New York at New Paltz New Paltz New York USA; ^3^ Translational Genomics Laboratory McLean Hospital Belmont Massachusetts USA; ^4^ Taub Institute for Research on Alzheimer's Disease and the Aging Brain Columbia University New York City New York USA

**Keywords:** aging, intergenerational inheritance, Lansing effect, life history, maternal effects, rotifers

## Abstract

Across diverse taxa, offspring from older mothers have decreased lifespan and fitness. Little is known about the extent to which maternal age effects vary among genotypes for a given species, however, except for studies of a few arthropod species. To investigate the presence and degree of intraspecific variability in maternal age effects, we compared lifespan, reproductive schedule, and lifetime reproductive output of offspring produced by young, middle‐aged, and old mothers in four strains of rotifers in the *Brachionus plicatilis* species complex. We found significant variability among strains in the magnitude and direction of maternal age effects on offspring life history traits. In one strain, offspring of young mothers lived 20% longer than offspring of old mothers, whereas there were no significant effects of maternal age on lifespan for other strains. Depending on strain, advanced maternal age had positive effects, negative effects, or no effect on lifetime reproductive output. Across strains, older mothers produced offspring that had higher maximum daily reproduction early in life. The effects of maternal age on offspring vital rates could not be explained by changes in trade‐offs between lifespan and reproduction. This study documents intraspecific variability in maternal age effects in an additional clade. Investigating intraspecific variability is critical for understanding the ubiquity of maternal age effects and their role in the evolution of life history and aging.

## Graphical abstract

We compared maternal age effects among four strains of rotifers in the *Brachionus plicatilis* species complex. For each strain, we measured lifespan, reproductive schedule, and lifetime reproductive output of offspring produced by young, middle‐aged, and old mothers. We found significant variability among strains in the magnitude and direction of maternal age effects on offspring life history traits.

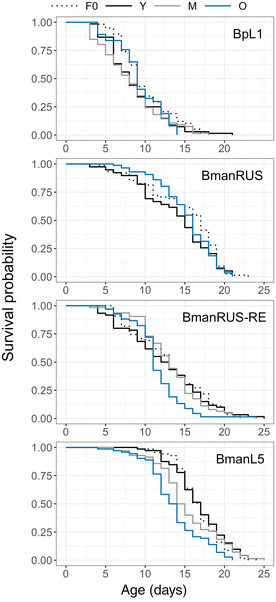

## 1 INTRODUCTION

Maternal age effects, in which a mother's age at the time of reproduction influences the phenotype of her offspring, are a commonly occurring form of intergenerational plasticity. In one of the earliest experimental studies of maternal age effects, Albert Lansing found that in the rotifer *Philodina citrina*, offspring lifespan and fecundity decreased in lineages reared from old mothers over multiple generations, while offspring from young mother lineages had long lifespan and high fecundity (Lansing, 1947, 1954). Thus, negative advanced maternal age effects are often referred to as “Lansing Effects.” Negative effects of advanced maternal age on offspring development, survival, fecundity, or health have been observed in many other taxa as well (Barclay & Myrskylä, 2016; Beamonte‐Barrientos et al., 2010; Bloch Qazi et al., 2017; Bock et al., 2019; Fox et al., 2003; Plaistow et al., 2015). In some species, however, advanced maternal age has positive effects (e.g., Krishna et al., 2012; Perez et al., 2017; Travers et al., 2021) or no apparent impact (e.g., Yılmaz et al., 2008) on offspring fitness.

Patterns of maternal age effects vary across taxonomically distantly related groups that have divergent morphologies, vital rates, and life histories. A meta‐analysis of 97 animal species showed that advanced maternal age has negative effects on offspring in some invertebrates, including *Caenorhabditis elegans*, rotifers, copepods, annelids, snails, fruit flies, and Lepidopterans; non‐agricultural mammals; and humans, whereas some wild birds exhibit positive maternal age effects on the early development of offspring (Ivimey‐Cook & Moorad, 2020). Differences among studies in the metrics assessed and findings for each taxonomic group highlight that maternal age effects are not uniformly positive or negative on all traits for a given species, however. For example, for *C. elegans*, advanced maternal age had positive effects on offspring reproduction and development in some studies, but caused shorter offspring lifespan in others (Perez et al., 2017; Travers et al., 2021).

The magnitude, direction, and mechanisms of maternal age effects could be expected to vary among distantly related taxa with different physiology, life history strategy, and evolutionary history. Studies of four arthropod species in three genera have demonstrated that maternal age effects also vary within a species, among strains with similar environmental niches, life cycles, and vital rates. Studies of *Daphnia* (Anderson et al., 2022; Plaistow et al., 2015), *Drosophila* (Bloch Qazi et al., 2017; Lee et al., 2019; Priest et al., 2002; Yılmaz et al., 2008), and crickets (Mousseau, 1991) provide examples of variability in the magnitude and direction of parental age effects on lifespan, development, fecundity, or embryonic diapause among strains and populations. Only one of these studies (Plaistow et al., 2015) analyzed offspring lifespan, development, and reproduction, while the other studies focused on a single response metric, typically lifespan, when comparing maternal age effects among strains. Investigation of multiple life history variables in additional, diverse species will allow us to better understand the range of intraspecific variability in maternal age effects.

Various mechanisms have been hypothesized to drive maternal age effects. Negative maternal age effects are thought to be caused by age‐related declines in reproductive system function or gamete quality, or by other physiological constraints, as mothers age (Lansing, 1947; Monaghan et al., 2020). Both positive and negative maternal age effects could be the result of age‐related life history adaptations, such as shifting of reproductive schedules across generations (Monaghan et al., 2020; Mousseau, 1991). Many have hypothesized that maternal‐age‐associated shifts in trade‐offs between offspring lifespan and reproduction are the source of maternal age effects. In keeping with life history theory and the related disposable soma hypothesis, if more resources are allocated to higher reproduction, then fewer resources are available to be allocated to somatic maintenance, repair, and growth, which could result in decreased lifespan, or vice versa (Kirkwood, 1977; Kirkwood et al., 1979; Stearns, 1976, 1989). For example, if older mothers provision fewer lipids, gene transcripts, organelles, metabolites, or other resources to developing offspring, those offspring may be shorter‐lived or have altered developmental and reproductive schedules. Such changes in early life reproductive schedule are thought to impact late‐life reproduction and lifespan (Stearns, 1976; Williams, 1957). Positive maternal age effects could occur in organisms with life histories, morphologies, or physiologies that enable parents to allocate more resources to offspring later in life (e.g., if older parents are larger or more experienced; Ivimey‐Cook & Moorad, 2020; Travers et al., 2021). Alternatively, higher resource allocation to offspring from older mothers could accelerate offspring development and reproduction, resulting in shorter lifespan (Plaistow et al., 2015). It remains poorly understood how maternal age effects on reproduction or on trade‐offs between lifespan and reproduction vary among species or strains.

Investigation of intraspecific variability in maternal age effects may provide insights into their evolution and mechanisms. Within a species, we can compare maternal age effects among populations or strains with relatively similar genetics, morphology, life history strategies, and environments, but with distinct vital rates or recent selective pressures. Additional examples across species will help to distinguish the effects and constraints of phylogeny and environment on maternal age effects. For example, variation in patterns of maternal age effects by genotype could suggest that maternal age effects are not caused only by age‐related accumulation of mutation or cellular damage in the maternal germline (e.g., Gao et al., 2019; Zhang et al., 2023), but rather may be modulated by underlying differences in genome sequence.

To broaden our understanding of intraspecific variability in maternal age effects in clades outside of the arthropods studied to date, and to investigate the role of trade‐offs between lifespan and reproduction as drivers of maternal age effects, we measured the effect of maternal age on offspring lifespan and reproduction in four strains of *Brachionus plicatilis* group rotifers. Rotifers are microscopic, aquatic invertebrates that have been used to study aging and maternal effects for over 80 years (King, 1969; King & Miracle, 1980; Ricci, 1983; Snell, 2014; Snell et al., 2006, 2015; Snell & Boyer, 1988; Wallace, 2002). As members of the Spiralia clade (Laumer et al., 2015), rotifers provide an evolutionarily distant comparison to the arthropod species (Ecdysozoa) in which intraspecific variability in maternal age effects have been examined thus far.

Species in the *Brachionus plicatilis* cryptic species complex (Gómez et al., 2002; Mills et al., 2007) have lifespans from 1 to 4 weeks and are easily reared across multiple, age‐synchronized generations in the laboratory (Gribble, 2021; Gribble & Snell, 2018). Maternal effects in response to inter‐ and intra‐specific interactions, including inducible defenses and the induction of sexual reproduction, have been studied in these rotifers for decades (Gilbert, 1966; Gilbert & McPeek, 2013; Snell & Boyer, 1988). *Brachionus* amictic females make a significant reproductive investment in each offspring, producing one to six large neonates each day throughout the reproductive period, with a lifetime reproductive output of 20–30 offspring. *Brachionus* species have externally brooded embryos, direct development with no larval stages, and no parental care after neonates hatch. Thus, we can measure lifespan and fecundity from birth in these species, without the confounding effects of metamorphoses or postnatal maternal care (Gribble, 2021; Gribble & Snell, 2018).

Here, we examined the effect of maternal age on offspring lifespan, lifetime reproductive output, and reproductive schedule in three strains of *B. manjavacas* and one strain of *B. plicatilis*. Closely‐related *Brachionus* strains can have large differences in lifespan and reproductive plasticity in response to environmental stressors (Gribble et al., 2018; Gribble, Kaido, et al., 2014; Snell, 1986), but the variability in maternal age effects among *Brachionus* strains is unknown. Previous studies on a single strain of *B. manjavacas* found that advanced maternal age leads to decreased lifespan and lifetime fecundity in offspring, but did not find evidence for maternal age‐related lifespan‐reproduction trade‐offs (Bock et al., 2019; Gribble, Jarvis, et al., 2014). We wanted to determine whether apparent trade‐offs were also lacking in other closely related strains. This work adds taxonomic breadth and analysis of additional variables to the existing examples of intraspecific variability in maternal age effects and will inform future research on the evolution and the mechanisms of maternal age effects within and across clades.

## 2 METHODS

### 2.1 Rotifer and phytoplankton culture

We studied one strain of *Brachionus plicatilis* (BpL1) and three strains of *B. manjavacas* (BmanL5, BmanRUS, and BmanRUS‐RE), which are all in the *B. plicatilis* cryptic species complex (Gómez et al., 2002; Mills et al., 2007). All strains except for BmanRUS‐RE have been maintained in constant culture for >15 years. The origins of these strains and their vital rates in the laboratory have been described elsewhere (Gribble et al., 2018; Gribble, Kaido, et al., 2014). BmanRUS‐RE was newly hatched from 5‐year‐old resting (diapausing) eggs of BmanRUS and was maintained in serial culture for 2 months before experimentation. We compared BmanRUS and BmanRUS‐RE to test whether the life history phenotypes that have been observed in past studies were preserved in the diapausing strain (BmanRUS‐RE), which had not been subjected to laboratory selective pressures for the past 5 years (Gribble et al., 2018; Gribble, Kaido, et al., 2014).

Prior to experimentation, each rotifer strain was kept in serial culture in 250 mL Erlenmeyer flasks containing 15 ppt Instant Ocean Sea Salt (Instant Ocean, Blacksburg, VA) in distilled water and ad libitum quantities of the chlorophyte *Tetraselmis suecica* (density of ~4 rotifers mL^−1^). Algae cultures were maintained in 2 L flasks of bubbled f/2 medium (Guillard, 1975) minus silica, also at a salinity of 15 ppt. Rotifer and algae stock cultures and experimental cohorts (described below) were maintained at 21°C on a 12:12 h light: dark cycle. Cultures of *T. suecica* were kept in semi‐continuous log phase growth by the removal of 40% of the culture and replacement with f/2 medium every other day throughout the experiments.

### 2.2 Life table experiments

The BmanRUS experiment took place in March 2021. The other three strains were studied simultaneously in July 2021. The same methods were used for all strains. To control for maternal and grandmaternal ages of the experimental animals, two generations of maternal age synchronization were conducted before the initiation of life table experiments (Figure S1). Amictic eggs were removed from mature females by vortexing, isolated by micropipette, and allowed to hatch and mature for 5 days in ad libitum food conditions, at which time eggs were again collected from mature females. After repeating this for two generations, eggs were collected and allowed to hatch for 6 h to initiate the F0 generation. Thus, the mothers and grandmothers of our experimental F0 cohorts were all 3–5 days old, and the F0 individuals were all of the same age ± 6 h.

To initiate and track each cohort, individual neonates were allocated to 1 mL of *T. suecica* at a concentration of 6 × 10^5^ cells mL^−1^ in 15 ppt Instant Ocean in 24‐well plates (*n* = 55 to 75 per strain). Every 24 h, each individual was observed on a Zeiss Stemi 508 microscope, and survival, reproductive status (carrying or not carrying eggs), and the number of new offspring were quantified. The original female was then transferred to a well with new seawater and *T. suecica*. Daily scoring and transfers were conducted until all individuals had died. This method produced individual‐level lifespan and reproduction data under highly controlled environmental conditions.

To initiate the F1 generation at young (Y), middle (M), and old (O) maternal ages for each strain (Figure S1), one neonate per mother (hatched within the previous 24 h) was pipetted into a well of a new plate with 1 mL of *T. suecica* in Instant Ocean. In some cases, multiple neonates were taken from a single mother, particularly if other mothers had no neonates on the day of collection. These F1 offspring were then tracked for their entire lifetimes in the same manner as their mothers. Because of the strains' varied lifespans and reproductive schedules, biological age and chronological age are not equivalent among strains. Based on preliminary studies, old maternal age for each strain was chosen as the day at which mean daily reproduction for F0 individuals dropped below one neonate per mother, to allow comparison of equivalent biological maternal ages among strains (Bock et al., 2019; Gribble et al., 2018; Gribble, Kaido, et al., 2014). For BmanL5 (*n* = 70–72 per maternal age cohort) and BmanRUS‐RE (*n* = 60–68), experimental F1 generations were initiated at maternal ages of 3 (Y), 6 (M), and 10 days (O). For BpL1, maternal ages were 3 (*n* = 68), 6 (*n* = 66), and 9 days (*n* = 37). Because multiple maternal ages have been previously studied in BmanRUS (Bock et al., 2019; Gribble, Jarvis, et al., 2014), we examined F1 cohorts from young (3 days; *n* = 39) and old (11 days; *n* = 86) maternal ages in this study.

### 2.3 Statistical analyses

All data were plotted and analyzed using R v. 4.0.2 (R Core Team, 2020). The following response metrics were calculated from lifespan and/or daily reproduction data: lifetime reproductive output (LRO), age at onset of reproduction, maximum daily reproduction (MDR), age of MDR, and reproductive period (as a percentage of lifespan). Intrinsic value, or the proportion of expected LRO remaining at a specific age (Dudycha, 2001), was calculated per day throughout the lifespan of each individual. From intrinsic value calculations, the age at which 50% of LRO was produced was determined per individual. The “survival” and “survminer” packages (Kassambara et al., 2017; Therneau, 2023) were used to create Kaplan–Meier survivorship curves.

For each response, a separate generalized linear model (GLM) was fit to test whether maternal age effects differ among strains. To model LRO (overdispersed count data) as a function of maternal age and strain (fixed categorical variables), a negative binomial GLM with a log link function was used, with an interaction term of *maternal age x strain*. To model lifespan, age at onset of reproduction, MDR, age of MDR, and age of 50% LRO (count data), a Poisson GLM with a log link function was used for each. To model reproductive period (proportion data), a binomial GLM with a logit link function was used. Since the reproductive period data were overdispersed, standard errors were corrected using a quasi‐GLM model. Analysis of deviance tests were conducted to determine whether the *maternal age x strain* interaction term contributed significantly to each model. The *X* ^*2*^ statistic is the difference in deviances between the full model and a reduced model, in which the interaction term was dropped. Analysis of deviance for the quasi‐GLM model uses the *F* statistic. For models with a significant interaction term, contrasts among maternal age cohorts within each strain were made using the “emmeans” package (Lenth, 2023), which obtains estimated marginal means for factor combinations. For models with only main effects of maternal age and/or strain (no significant interaction), contrasts were made among levels of each factor, while pooling levels of the other factor.

To test for life history trade‐offs, linear models were fit to describe the relationships between lifespan and each of LRO, reproductive period, MDR, and the age of 50% LRO. For each strain, analysis of covariance was conducted to determine whether the slopes of these relationships differed among maternal age cohorts (indicated by a significant interaction between maternal age cohort and lifespan).

## 3 RESULTS

### 3.1 Lifespan

**FIGURE 1:**
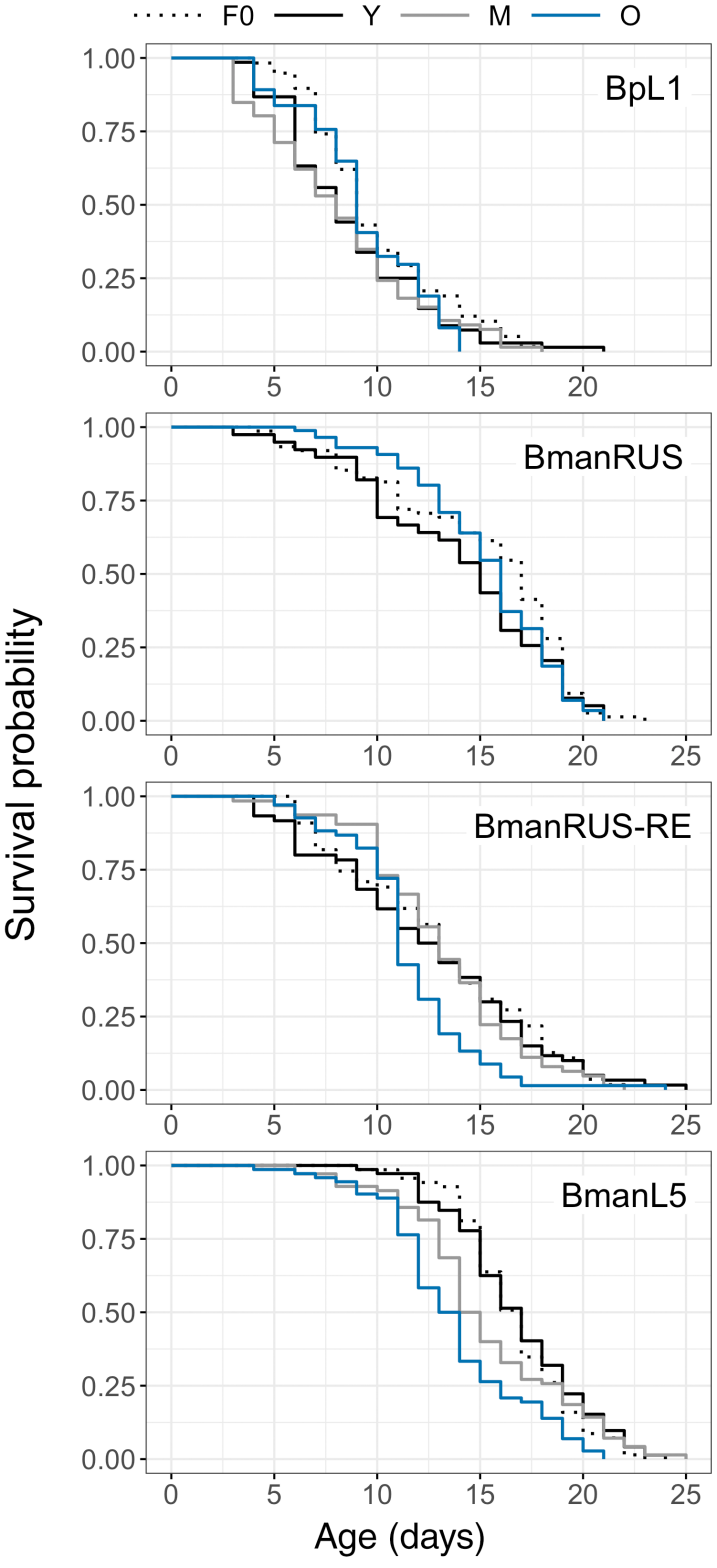
Survivorship curves of the F0 generation and of the F1 generation from young (Y), middle‐aged (M), and old (O) mothers, for four *Brachionus* strains.

Maternal age effects on offspring lifespan differed among strains (Figure 1; significant *maternal age x strain* interaction: *X* ^2^(5) = 29.7, *p* < .0001). For BmanL5, rotifers in both the Y and M cohorts had longer lifespans than those of the O cohort, by 3.1 and 1.6 days on average, respectively. For BmanRUS‐RE, M cohort rotifers had longer lifespans than those of the O cohort by 1.6 days on average. There were no significant differences among maternal age cohorts for BmanRUS and BpL1 (Table 1 and Table S1). Maximum lifespan (age of 5% survivorship) was shorter for O cohort rotifers in all strains (Figure 1; Table S1).

**TABLE 1:** Results of post hoc contrasts of estimated marginal means, calculated from generalized linear models that were fit for each response metric.

Contrast	Estimate	SE	*z*‐ratio	*p*‐value
Lifespan (significant *maternal age x strain* interaction)				
BpL1				
Y – M	0.05	0.06	0.87	.660
Y – O	−0.07	0.07	−1.05	.546
M – O	−0.12	0.07	−1.78	.175
BmanL5				
Y – M	0.09	0.04	2.26	.062
Y – O	0.20	0.04	4.75	**<.0001**
M – O	0.11	0.04	2.47	**.036**
BmanRUS				
Y – O	−0.09	0.05	−1.79	.073
BmanRUS‐RE				
Y – M	−0.05	0.05	−1.00	.577
Y – O	0.08	0.05	1.58	.256
M – O	0.13	0.05	2.63	**.023**
LRO (significant *maternal age x strain* interaction)				
BpL1				
Y – M	−0.24	0.06	−3.89	**.0003**
Y – O	−0.19	0.07	−2.60	**.03**
M – O	0.05	0.07	0.72	.75
BmanL5				
Y – M	0.11	0.04	2.66	**.022**
Y – O	0.11	0.04	2.62	**.024**
M – O	−0.002	0.04	−0.05	.998
BmanRUS				
Y – O	−0.06	0.05	−1.26	.208
BmanRUS‐RE				
Y – M	−0.08	0.04	−1.89	.142
Y – O	−0.11	0.04	−2.58	**.027**
M – O	−0.03	0.04	−0.67	.784
MDR (significant main effects)				
Maternal age				
Y – M	−0.15	0.05	−2.84	**.013**
Y – O	−0.18	0.05	−3.79	**.0004**
M – O	−0.04	0.05	−0.74	.743
Strain				
BpL1 – L5	−0.57	0.06	−9.83	**<.0001**
BpL1 – RUS	−0.18	0.07	−2.42	.073
BpL1 – RUS‐RE	−0.48	0.06	−7.95	**<.0001**
L5 – RUS	0.39	0.06	6.28	**<.0001**
L5 – RUS‐RE	0.09	0.05	1.94	.213
RUS – RUS‐RE	−0.30	0.06	−4.67	**<.0001**
Rep. period (significant *maternal age x strain* interaction)				
BpL1				
Y – M	−0.40	0.13	−3.01	**.007**
Y – O	−0.04	0.15	−0.28	.958
M – O	0.36	0.16	2.33	.05
BmanL5				
Y – M	0.29	0.09	3.10	**.0056**
Y – O	0.40	0.10	4.18	**.0001**
M – O	0.11	0.10	1.12	.499
BmanRUS				
Y – O	0.53	0.14	3.81	**.0001**
BmanRUS‐RE				
Y – M	0.13	0.13	0.99	.585
Y – O	0.25	0.13	1.92	.134
M – O	0.12	0.12	0.97	.597

*Note*: For lifespan, lifetime reproductive output (LRO), and reproductive period (% of the lifespan), *maternal age x strain* interaction terms contributed significantly, thus contrasts were conducted among maternal age cohorts within each strain. For maximum daily reproduction (MDR), there was a significant main effect of maternal age (no significant interaction), thus contrasts were conducted among levels of each factor, while pooling levels of the other factor. Results of contrasts for response metrics for which there were no significant effects of maternal age or interactions are shown in Table S2. Maternal age cohorts: Y = young mothers, M = middle‐aged mothers, O = old mothers. Significant *p*‐values were bolded (*p* < .05).

### 3.2 Lifetime reproductive output

**FIGURE 2:**
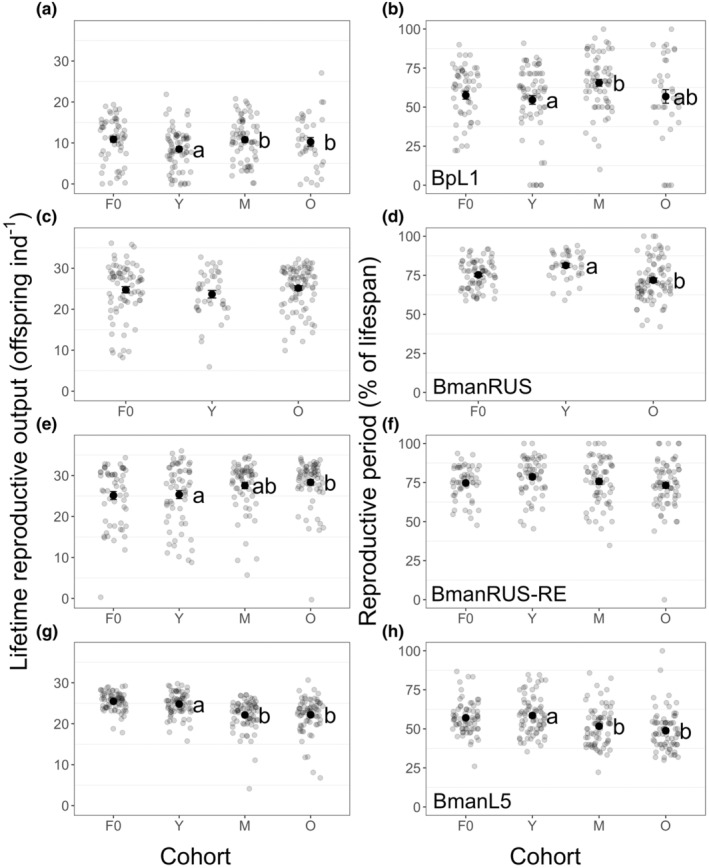
Lifetime reproductive output (LRO; a, c, e, g) and reproductive period (percent of the lifespan spent carrying eggs; b, d, f, h) for the BpL1 (a, b), BmanRUS (c, d), BmanRUS‐RE (e, f), and BmanL5 (g, h) strains. Means for the F0 generation and young (Y), middle‐aged (M), and old (O) mother cohorts of the F1 generation are shown by bold, black points (±SE) and individual data points are shown in gray. Significant differences among cohorts within strains are indicated by letters to the right of the mean points (contrasts of estimated marginal means, alpha = .05; note the F0 generation was excluded from analyses).

Maternal age effects on lifetime reproductive output (LRO) differed among strains (Figure 2; significant *maternal age x strain* interaction: *X* ^2^(5) = 31.2, *p* < .0001). For BmanL5, Y cohort rotifers had higher LRO than did those of the M and O cohorts (Table 1 and Table S1; Figure 2g). For BpL1 and BmanRUS‐RE, the opposite effect occurred: Y cohort rotifers had lower LRO than did older maternal age cohorts (Table 1 and Table S1; Figure 2a,e). For BmanRUS, there was no significant effect of maternal age on LRO (Table 1 and Table S1; Figure 2c). Variance in LRO among individuals differed significantly among maternal age cohorts for BmanRUS‐RE (Y = 58.3, M = 37.8, O = 19.2; Levene's test: *F*(2,188) = 5.37, *p* = .005). For the other strains, variances in LRO were not significantly different among maternal age cohorts (Table S1).

### 3.3 Timing of reproduction

**FIGURE 3:**
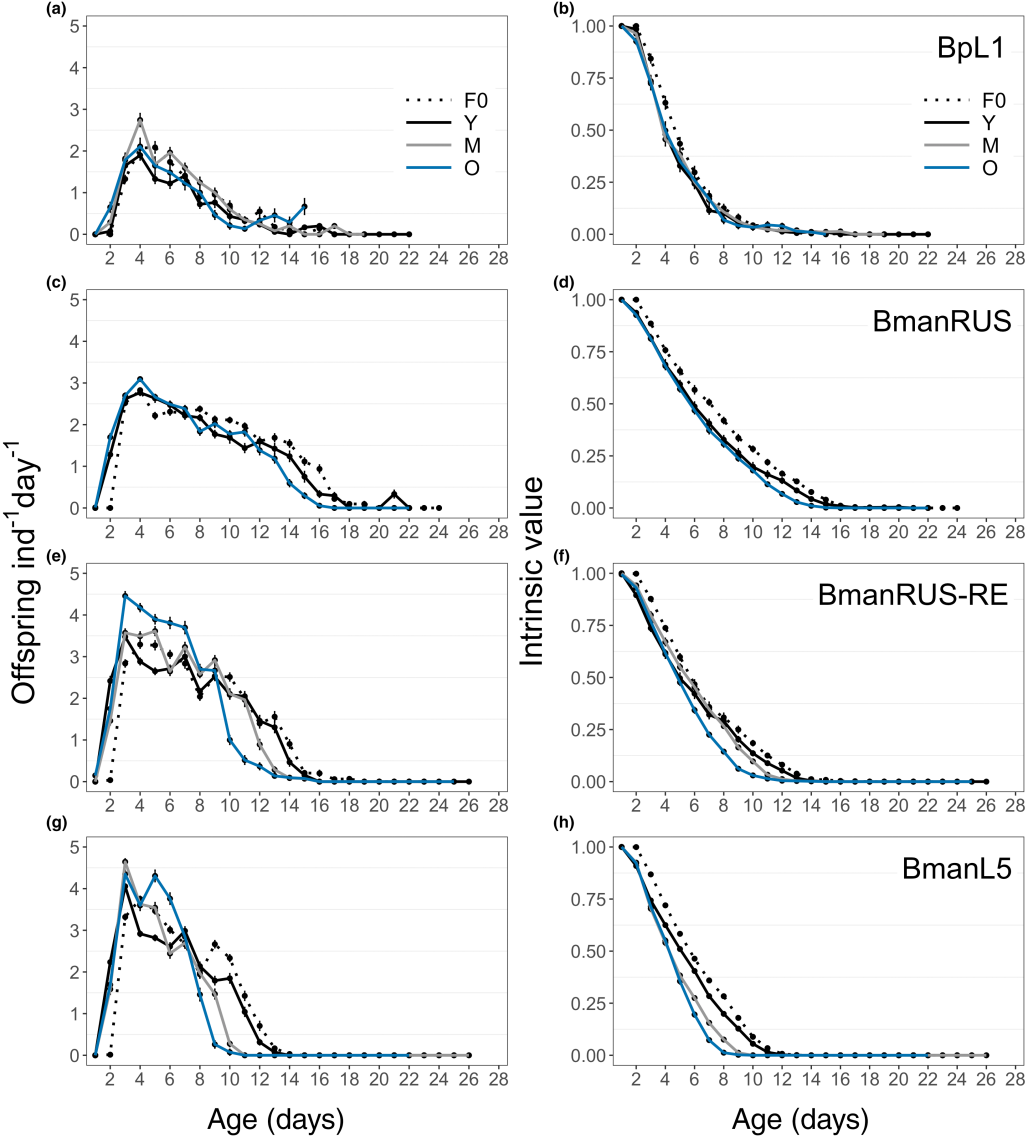
Daily reproduction (a, c, e, g) and intrinsic value (b, d, f, h) for the BpL1 (a, b), BmanRUS (c, d), BmanRUS‐RE (e, f), and BmanL5 (g, h) strains. Daily mean values are shown by bold points (±SE), and young (Y), middle‐aged (M), and old (O) mother cohorts are indicated by line color.

Across strains, O and M cohort rotifers had higher maximum daily reproduction (MDR) than did Y cohort rotifers (Table 1 and Table S1). There was no effect of maternal age on the age at which MDR occurred, although there were significant differences among strains (Table S1 and Table S2). The age by which rotifers had produced 50% of their LRO also significantly differed among strains, but not among maternal ages (Table S1 and Table S2). Across strains, the majority of individuals started producing neonates on at two days old, except for BpL1, which had more variability in the onset of reproduction (no significant effect of maternal age, significant strain effect; Table S2). Daily reproductive output and intrinsic value are summarized over time in Figure 3.

Reproductive period, measured as the percent of the lifespan during which mothers carried eggs, was affected by maternal age in different ways among strains (Figure 2; significant *maternal age x strain* interaction term: *F*(5) = 3.98, *p* = .001). For BmanL5 and BmanRUS, rotifers in the Y cohort had longer reproductive periods than those in older cohorts (Figure 2d,h). For BpL1, the longest reproductive periods were observed in the M cohort (Figure 2b). Reproductive periods did not significantly differ among maternal age cohorts for BmanRUS‐RE (Figure 2f; Table 1 and Table S1).

### 3.4 Life history trade‐offs

**FIGURE 4:**
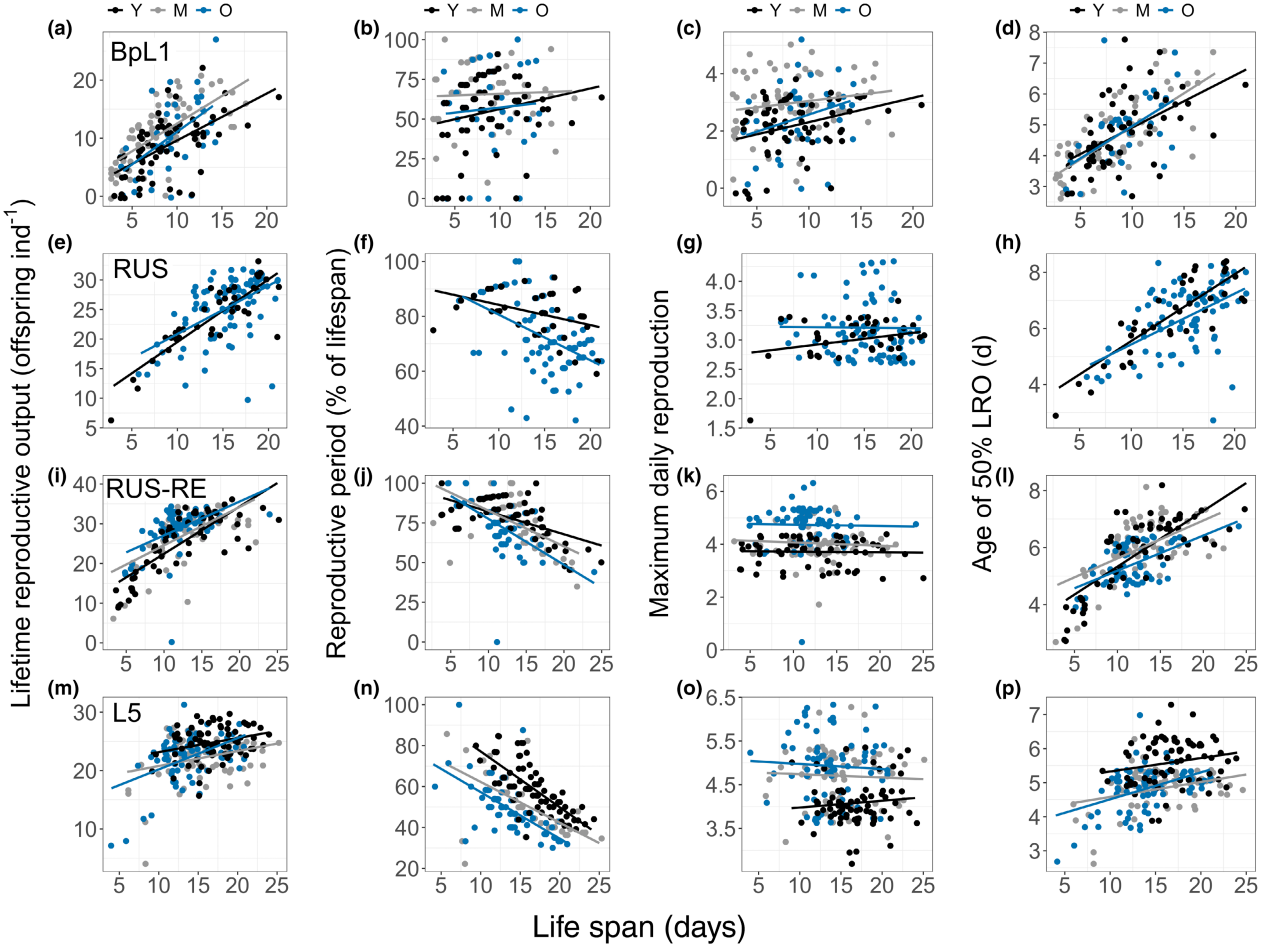
Relationships between lifespan and lifetime reproductive output (LRO; a, e, i, m), reproductive period (as a percent of the lifespan; b, f, j, n), maximum daily reproduction (MDR; c, g, k, o), and age of 50% LRO in days (d, h, l, p) for the BpL1 (a–d), BmanRUS (e–h), BmanRUS‐RE (i–l), and BmanL5 (m–p) strains. Young (Y), middle‐aged (M), and old (O) mother cohorts are indicated within each strain by point and line color. Note differences between strains in scales of *y*‐axes.

Relationships between lifespan and each of four metrics of reproduction differed among strains, but there were few significant differences in these correlations among maternal age cohorts within strains. LRO increased with lifespan across all strains. These lifespan:LRO correlations were not significantly different among maternal age cohorts within any strain (Table 2; Figure 4). Reproductive period (as a percentage of lifespan) decreased with increasing lifespan across strains, except for BpL1, for which there was no significant relationship (Table 2). For BmanRUS‐RE, the negative slope of the relationship was steepest in the O cohort versus younger cohorts (Table 2; Figure 4j). There were no significant correlations between MDR and lifespan across strains and maternal age cohorts (Table 2; Figure 4). The age of 50% LRO increased with lifespan across strains. For BmanRUS‐RE, the slope of this relationship was significantly greater in the Y cohort than other cohorts (Table 2; Figure 4l). Correlations did not significantly differ among maternal age cohorts in the other strains (Table 2; Figure 4d,h,p).

**TABLE 2:** Results of analysis of covariance of trendlines of lifespan versus each of four metrics of reproduction: lifetime reproductive output (LRO), reproductive period, maximum daily reproduction (MDR), and the age of 50% LRO (d).

	Df	Sum sq	Mean sq	*F*‐value	*p*‐value
LRO L5					
Lifespan	1	495.6	495.6	43.05	**<.0001**
Maternal age	2	165.9	83.0	7.20	**.0009**
Lifespan: maternal age	2	51.0	25.5	2.21	.112
Residuals	208	2394.8	11.5	NA	NA
LRO RUS					
Lifespan	1	1636.1	1636.1	98.20	**<.0001**
Maternal age	1	1.8	1.8	0.11	.7429
Lifespan: maternal age	1	26.6	26.6	1.59	.2091
Residuals	121	2016.0	16.7	NA	NA
LRO RUS‐RE					
Lifespan	1	3255.4	3255.4	139.00	**<.0001**
Maternal age	2	519.4	259.7	11.09	**<.0001**
Lifespan: maternal age	2	61.5	30.8	1.31	.271
Residuals	185	4332.8	23.4	NA	NA
LRO BpL1					
Lifespan	1	1816.5	1816.5	96.43	**<.0001**
Maternal age	2	244.5	122.3	6.49	**.0019**
Lifespan: maternal age	2	24.8	12.4	0.66	.5196
Residuals	165	3108.3	18.8	NA	NA
Rep. period L5					
Lifespan	1	9128.2	9128.2	108.93	**<.0001**
Maternal age	2	9246.1	4623.1	55.17	**<.0001**
Lifespan: maternal age	2	249.8	124.9	1.49	.2276
Residuals	208	17,430.2	83.8	NA	NA
Rep. period RUS					
Lifespan	1	3700.7	3700.7	32.56	**<.0001**
Maternal age	1	1573.7	1573.7	13.85	**.0003**
Lifespan: maternal age	1	388.2	388.2	3.42	.067
Residuals	121	13,752.5	113.7	NA	NA
Rep. period RUS‐RE					
Lifespan	1	11,184.8	11,184.8	77.56	**<.0001**
Maternal age	2	1873.7	936.9	6.50	**.0019**
Lifespan: maternal age	2	1077.4	538.7	3.74	**.0257**
Residuals	185	26,677.7	144.2	NA	NA
Rep. period BpL1					
Lifespan	1	792.3	792.3	1.65	.200
Maternal age	2	4647.1	2323.5	4.85	**.009**
Lifespan: maternal age	2	553.6	276.8	0.58	.562
Residuals	165	79,020.8	478.9	NA	NA
MDR L5					
Lifespan	1	3.2	3.2	9.24	**.0027**
Maternal age	2	24.4	12.2	35.00	**<.0001**
Lifespan: maternal age	2	0.4	0.2	0.51	.601
Residuals	208	72.5	0.3	NA	NA
MDR RUS					
Lifespan	1	0.3	0.3	1.93	.168
Maternal age	1	1.0	1.0	7.86	**.0059**
Lifespan: maternal age	1	0.2	0.2	1.55	.216
Residuals	121	15.9	0.13	NA	NA
MDR RUS‐RE					
Lifespan	1	1.2	1.2	3.21	.075
Maternal age	2	34.2	17.1	47.05	**<.0001**
Lifespan: maternal age	2	0.1	0.03	0.07	.930
Residuals	185	67.2	0.4	NA	NA
MDR BpL1					
Lifespan	1	9.5	9.5	9.50	**.0024**
Maternal age	2	22.5	11.3	11.28	**<.0001**
Lifespan: maternal age	2	1.7	0.8	0.83	.439
Residuals	165	164.6	1.0	NA	NA
50% LRO L5					
Lifespan	1	18.2	18.2	53.95	**<.0001**
Maternal age	2	19.7	9.83	29.18	**<.0001**
Lifespan: maternal age	2	0.85	0.42	1.26	.2858
Residuals	208	70.1	0.34	NA	NA
50% LRO RUS					
Lifespan	1	75.0	75.0	88.80	**<.0001**
Maternal age	1	3.75	3.75	4.44	**.037**
Lifespan: maternal age	1	1.47	1.47	1.75	.189
Residuals	121	102.2	0.84	NA	NA
50% LRO RUS‐RE					
Lifespan	1	91.6	91.6	173.05	**<.0001**
Maternal age	2	5.33	2.66	5.03	**.0074**
Lifespan: maternal age	2	3.50	1.75	3.31	**.0388**
Residuals	183	96.8	0.53	NA	NA
50% LRO BpL1					
Lifespan	1	80.7	80.7	99.53	**<.0001**
Maternal age	2	0.02	0.01	0.01	.9868
Lifespan: maternal age	2	0.85	0.42	0.52	.5936
Residuals	154	124.8	0.81	NA	NA

*Note*: The trendlines of maternal age cohorts were contrasted within each strain. A *lifespan: maternal age* interaction term with *p* < .05 indicates that slopes of the trendlines were significantly different. Significant *p*‐values were bolded (*p* < .05).

## 4 DISCUSSION

Maternal age effects are known to vary among species, in a manner thought to depend on large differences in life history strategies among evolutionarily distant taxa. Here we show intraspecific differences in the magnitude and direction of maternal age effects on offspring lifespan and reproduction in *Brachionus* rotifers. To our knowledge, this is the first examination of intraspecific variability in maternal age effects in a Spiralian species. Our finding that maternal age effects were genotype‐specific supports what has been found in arthropod studies, and suggests that maternal age effects are genetically determined traits which may differ among closely related species with identical life history strategies. These results suggest that maternal age effects are not simply caused by the age‐related accumulation of cellular or DNA damage that is passed on to offspring, as has been hypothesized (Gao et al., 2019; Zhang et al., 2023). Alternatively, if such damage accumulation does occur, some genotypes may have the capacity to better prevent or repair resulting dysfunction in the germline.

Our findings recapitulate what has been found previously in other species and add to our understanding of intraspecific diversity in maternal age effects on reproduction and lifespan. Intraspecific variability in maternal age effects has been described in a few arthropod species, with most studies conducted on *Drosophila melanogaster* and focused on lifespan effects. A comparison of six lab strains (four inbred and two outbred) of *D. melanogaster* showed that older mothers generally produced offspring with shorter lifespans, but in a single strain, Canton‐S, advanced maternal age led to longer lifespan in offspring (Priest et al., 2002). Lee et al. (2019) replicated the positive effect of advanced maternal age on offspring lifespan in Canton‐S and found negative maternal age effects in two additional strains. Bloch Qazi et al. (2017) studied Canton‐S and Oregon‐R and found that embryo viability (hatching success) and embryo to adult viability were lower in offspring of old mothers, even in the strain with positive effects of advanced maternal age on offspring lifespan, though the magnitude of effects differed between the strains. Three *D. melanogaster* populations collected from distinct environments in Turkey also showed both positive and negative effects of advanced maternal age on offspring longevity (Yılmaz et al., 2008). In contrast, we did not observe significantly increased lifespan in old‐mother offspring in any rotifer strain in the current study. Given the many differences in the experimental designs, species and strains used, ages sampled, and metrics measured between prior studies of intraspecific diversity in maternal age effects, we are unable to attribute differences between our findings and previous experiments to any specific experimental variable.

Rotifers and *Daphnia*, another arthropod in which intraspecific diversity in maternal age effects has been studied, are evolutionarily distant but have similar lifestyles and inhabit the same aquatic ecological niche. Thus, they might be expected to be under similar selective pressures that could affect maternal age effect phenotypes. In *Daphnia pulex*, advanced maternal age decreased offspring lifespan in two of three clones studied, but advanced maternal age led to higher growth rates in offspring in all three clones (Plaistow et al., 2015). In *Daphnia magna*, advanced maternal age had positive, negative, or no effect on lifespan across five clones (Anderson et al., 2022). Studies in other taxa are needed to distinguish the possible effects of phylogeny versus habitat in shaping maternal age effects.

Variability in the direction and magnitude of maternal age effects between species has been attributed to differences in life history strategy that result from varied selective pressures and evolutionary constraints. Intraspecific differences in maternal effects could also be due to differential selective pressures in diverse environments. For example, in the striped ground cricket, *Allonemobius fasciatus*, populations that span a wide latitudinal cline have evolved distinct life histories. Northern populations exposed to short growing seasons reproduce once per year (univoltine), while southern populations with longer growing seasons can reproduce multiple times per year (multivoltine). In univoltine populations, maternal age had no effect on the tendency for diapause in offspring. At the end of the short growing season, mating adults produce eggs that diapause, overwinter, and hatch in the spring with sufficient time for maturation. In multivoltine populations, the tendency to produce diapausing eggs increased with maternal age. This is advantageous, since the probability of producing offspring that will mature before winter decreases as the growing season progresses (Mousseau, 1991).

The strains studied here were originally isolated from different geographic sites (Gribble, Kaido, et al., 2014), but all have been continuously cultured in the lab under similar serial culture methods for decades. These strains have been subjected to the same conditions of no predation, a consistent daily light: dark cycle, constant temperature, cycles of high and low food, cycles of high and low population density, and frequent population bottlenecks. Therefore, given generation times of 2–4 days, all strains have been exposed to similar selective pressures for thousands of generations. Differences in initial genetic composition at the time of culture origin, spontaneous mutation, and genetic drift caused by frequent population bottlenecks all may have contributed to the laboratory evolution of these strains, which could underlie the variation in vital rates and maternal age effects observed here.

In previous BmanRUS studies, offspring of old mothers had shorter lifespan than offspring of young mothers by 10%–25% and lower LRO by ~50% (Bock et al., 2019; Gribble, Jarvis, et al., 2014). Here, maternal age had no significant effect on lifespan or LRO in BmanRUS. We expected that BmanRUS‐RE initiated from five‐year‐old BmanRUS resting eggs would be genetically and phenotypically more similar to BmanRUS used in previous studies than to the current continuously cultured BmanRUS strain, due to possible recent laboratory evolution. In BmanRUS‐RE, M cohort rotifers lived an average of 1.6 days longer than O cohort rotifers. Offspring of older mothers had *higher* LRO than those of young mothers. Thus, the previously observed strong negative maternal age effects in BmanRUS were only partially recovered in BmanRUS‐RE, possibly due to population bottlenecks and subsequent genetic differentiation.

Life history theory predicts a trade‐off between lifespan and reproduction, at least under limiting resources (Cohen et al., 2020). In the biology of aging literature, this is often interpreted to mean that increased reproduction causes shorter lifespan or that limiting or delaying reproduction can increase longevity, and that maternal age effects are driven by such trade‐offs (Kirkwood, 1977; Kirkwood et al., 1979; Stearns, 1976, 1989). For example, Plaistow et al. (2015) attributed the negative lifespan effects of advanced maternal age to earlier reproduction by old mother offspring. In *Daphnia pulex*, they found that advanced maternal age led to shorter lifespan in two of three strains studied. Across all three strains, offspring of older mothers had larger clutch sizes earlier in life and faster growth than offspring of young mothers. Only in the two strains in which lifespan was reduced in offspring of old mothers did reproductive maturation occur at larger body sizes. Thus, the authors concluded that increased growth rate and early life reproduction traded‐off with lifespan and that maternal age effects were a result of better offspring provisioning by older mothers. Similarly, Anderson et al. (2022) studied multiple strains of *Daphnia magna* and found an “inverse Lansing Effect” in some strains, in which offspring of older mothers lived longer than offspring of younger mothers. They hypothesized that this inverse effect could be a result of *decreased* lipid offspring provisioning by older mothers, which could result in embryonic caloric restriction. The notion of a trade‐off between lifespan and reproduction also underlies this hypothesis: decreased lipid stores during development ultimately limits reproduction and subsequently increases lifespan. We did not find evidence of tradeoffs between lifespan and reproduction or reproductive schedule. This was reflected in our finding that in all strains, intrinsic value was identical among maternal age cohorts in early life (up to age of 4 days), but was lower in old‐mother offspring in late life in two of four strains (BmanRUS‐RE, BmanL5), regardless of the direction of lifespan and fecundity changes.

Maternal age effects on offspring lifespan and reproduction are genotype‐specific in *Brachionus* rotifers. More studies examining intraspecific variability in maternal age effects are needed to determine whether such high variation is widespread among clades or is specific to arthropods and rotifers (the most well‐studied taxa to date). Future work on maternal age effects in rotifers and other species should include more genotypes, ideally spanning diverse life history strategies, ecological niches, and phylogenetic diversity. For example, comparing multiple strains with a range of tendencies for mixis and varied vital rates could allow for direct tests of the influence of life history traits on maternal age effects. Future quantitative trait locus (QTL) analyses or comparative genomics studies of strains with varied maternal age effects may provide insight into the genetic controls of intergenerational inheritance.

## AUTHOR CONTRIBUTIONS

**Alyssa Liguori:** Conceptualization (equal); data curation (equal); formal analysis (equal); investigation (equal); methodology (equal); project administration (equal); supervision (equal); visualization (equal); writing – original draft (equal); writing – review and editing (equal). **Sovannarith Korm:** Investigation (equal); writing – review and editing (equal). **Alex Profetto:** Investigation (equal); writing – review and editing (equal). **Emily Richters:** Investigation (equal); writing – review and editing (equal). **Kristin E. Gribble:** Conceptualization (equal); funding acquisition (equal); investigation (equal); methodology (equal); project administration (equal); resources (equal); supervision (equal); writing – original draft (equal); writing – review and editing (equal).

## CONFLICT OF INTEREST STATEMENT

The authors declare no conflict of interest.

## Supporting information


Data S1:

## Data Availability

The original data are available at DOI: 10.5061/dryad.b2rbnzspp, https://github.com/aliguori19/Brachionus_intraspecific_variation
