# Supplementary material for: Maternal age effects on offspring lifespan and reproduction vary within a species
Source: Ecol Evol. 2024 May 16;14(5):e11287. doi: 10.1002/ece3.11287 (PMC11097000; doi:10.1002/ece3.11287)
Supplement: Supplementary file 1 — Data S1: [file ECE3-14-e11287-s001.docx]

**Supplementary Material to:**

Maternal age effects on offspring lifespan and reproduction vary within a species

**Authors**: Alyssa Liguori^1,2^, Sovannarith Korm^1^, Alex Profetto^1,3^, Emily Richters^1,4^, Kristin E. Gribble^1^

^1^ Josephine Bay Paul Center for Comparative Molecular Biology and Evolution, Marine Biological Laboratory, Woods Hole, MA 02543

^2^ Department of Biology, State University of New York at New Paltz, New Paltz, NY 12561

^3^ Translational Genomics Laboratory, McLean Hospital, Belmont, MA 02478

^4^ Taub Institute for Research on Alzheimer’s Disease and the Aging Brain, Columbia University, New York, NY 10032

**Corresponding authors**: Alyssa Liguori and Kristin E. Gribble

Emails: [liguoria@newpaltz.edu](mailto:liguoria@newpaltz.edu), kgribble@mbl.edu

Mailing address: Marine Biological Laboratory, 7 MBL Street, Woods Hole, MA 02543

**Figure S1.** Experimental design across four generations (2 generations of age synchronization and 2 experimental generations). The mothers and grandmothers of our experimental F0 cohorts were all 3 – 5 days old. Young mother cohorts (Y; black) were initiated with 3-d-old mothers and middle-aged cohorts (M; gray) were initiated with 6-d-old mothers for all strains. Old mother cohorts (O; blue) were initiated with 9-d-old mothers for BpL1, 10-d-old mothers for BmanL5 and BmanRUS-RE, and 11-d-old mothers for BmanRUS.

**Table S1.** Summary statistics for lifespan and reproduction response metrics. LRO = lifetime reproductive output (in number of neonates). MDR = maximum daily reproduction (in number of neonates). SEM = standard error of the mean. The unit of time for lifespan and age is days.

| **Strain** | **Mat. age** | **n** | **Mean lifespan(SEM)** | **Median lifespan** | **Max lifespan (95th %tile)** | **Mean LRO (SEM)** | **Median LRO** | **Var. LRO** | **Min LRO** | **Max LRO** | **Mean MDR** | **Mean rep. period % (SEM)** | **Mean rep. period days (SEM)** | **Mean age of MDR** | **Mean age of 50% LRO** |  |
| --- | --- | --- | --- | --- | --- | --- | --- | --- | --- | --- | --- | --- | --- | --- | --- | --- |
|  |  |  |  |  |  |  |  |  |  |  |  |  |  |  |  |  |
|  |  |  |  |  |  |  |  |  |  |  |  |  |  |  |  |  |
| **Bman**  **L5** | 3 | 72 | 16.8 (0.39) | 17 | 22 | 24.8  (0.33) | 25 | 7.71 | 16 | 30 | 4.08 | 58.6  (1.43) | 10.1  (0.17) | 3.0 | 5.6 |  |
|  | 6 | 70 | 15.3 (0.49) | 14.5 | 22 | 22.1  (0.46) | 23 | 14.6 | 4 | 27 | 4.7 | 51.7  (1.5) | 8.1  (0.18) | 3.1 | 4.8 |  |
|  | 10 | 72 | 13.7 (0.42) | 13.5 | 20 | 22.2  (0.49) | 23 | 17.2 | 7 | 31 | 4.93 | 48.8  (1.48) | 6.9  (0.17) | 3.8 | 4.8 |  |
|  |  |  |  |  |  |  |  |  |  |  |  |  |  |  |  |  |
| **Bman**  **RUS** | 3 | 39 | 13.9 (0.74) | 15 | 21 | 23.7  (0.92) | 25 | 33.1 | 6 | 33 | 3 | 81.3  (1.38) | 12  (0.55) | 3.4 | 6.5 |  |
|  | 11 | 86 | 15.3 (0.37) | 16 | 20 | 25.2  (0.57) | 27 | 27.8 | 10 | 32 | 3.21 | 71.9  (1.39) | 11.5  (0.26) | 3.3 | 6.4 |  |
|  |  |  |  |  |  |  |  |  |  |  |  |  |  |  |  |  |
| **Bman**  **RUS-RE** | 3 | 60 | 12.4 (0.67) | 12.5 | 20 | 25.4  (0.99) | 27.5 | 58.3 | 9 | 36 | 3.72 | 78.6  (1.6) | 10.2  (0.41) | 3.5 | 5.8 |  |
|  | 6 | 63 | 13.1 (0.48) | 13 | 20 | 27.6  (0.78) | 30 | 37.8 | 6 | 35 | 4.03 | 75.7  (1.88) | 10.3  (0.29) | 3.7 | 6 |  |
|  | 10 | 68 | 11.5 (0.38) | 11 | 16 | 28.3  (0.68) | 30 | 19.2 | 0 | 34 | 4.74 | 73.2  (1.93) | 8.9  (0.24) | 3.5 | 5.4 |  |
|  |  |  |  |  |  |  |  |  |  |  |  |  |  |  |  |  |
| **BpL1** | 3 | 68 | 8.6 (0.44) | 8 | 15 | 8.5  (0.61) | 8.5 | 24.9 | 0 | 22 | 2.19 | 54.6  (2.76) | 5.4  (0.36) | 3.9 | 4.7 |  |
|  | 6 | 66 | 8.2 (0.48) | 8 | 16 | 10.8  (0.66) | 10.5 | 28.9 | 0 | 21 | 2.97 | 65.6  (2.19) | 6.1  (0.37) | 3.8 | 4.6 |  |
|  | 9 | 37 | 9.3 (0.50) | 9 | 14 | 10.3  (1.05) | 9 | 40.6 | 0 | 27 | 2.49 | 56.8  (4.36) | 5.9  (0.55) | 3.8 | 4.8 |  |

**Table S2.** Results of *post hoc* contrasts of estimated marginal means, calculated from generalized linear models that were fit for each response metric. Contrasts were conducted among levels of each factor, while pooling levels of the other factor, since there were no significant interactions for these response metrics. Maternal age cohorts: Y = young mothers, M = middle-aged mothers, O = old mothers. MDR = maximum daily reproduction, LRO = lifetime reproductive output.

|  | **Age of onset of reproduction (significant main effect of strain)** | | | |
| --- | --- | --- | --- | --- |
| **contrast** | **estimate** | **SE** | **z-ratio** | **p-value** |
| mat. age: Y - M | -0.01 | 0.06 | -0.20 | 0.977 |
| Y - O | 0.02 | 0.06 | 0.36 | 0.932 |
| M - O | 0.04 | 0.07 | 0.53 | 0.859 |
| strain: BpL1 – L5 | 0.27 | 0.07 | 4.01 | **0.0004** |
| BpL1 – RUS | 0.29 | 0.08 | 3.41 | **0.004** |
| BpL1 – RUS-RE | 0.32 | 0.07 | 4.53 | **< 0.0001** |
| L5 – RUS | 0.02 | 0.08 | 0.21 | 0.997 |
| L5 – RUS-RE | 0.05 | 0.07 | 0.70 | 0.897 |
| RUS – RUS-RE | 0.03 | 0.08 | 0.38 | 0.982 |
|  | **Age of MDR (significant main effect of strain)** | | | |
| mat. age: Y - M | -0.007 | 0.05 | -0.13 | 0.990 |
| Y - O | -0.06 | 0.05 | -1.17 | 0.470 |
| M - O | -0.05 | 0.05 | -0.95 | 0.612 |
| strain: BpL1 – L5 | 0.14 | 0.06 | 2.60 | **0.046** |
| BpL1 – RUS | 0.16 | 0.07 | 2.30 | 0.098 |
| BpL1 – RUS-RE | 0.08 | 0.06 | 1.42 | 0.488 |
| L5 – RUS | 0.01 | 0.06 | 0.18 | 0.998 |
| L5 – RUS-RE | -0.06 | 0.05 | -1.20 | 0.627 |
| RUS – RUS-RE | -0.08 | 0.06 | -1.18 | 0.642 |
|  | **Age of 50% LRO (significant main effect of strain)** | | | |
| mat. age: Y - M | 0.04 | 0.04 | 0.97 | 0.599 |
| Y - O | 0.07 | 0.04 | 1.75 | 0.186 |
| M - O | 0.03 | 0.04 | 0.62 | 0.810 |
| strain: BpL1 – L5 | -0.08 | 0.05 | -1.78 | 0.283 |
| BpL1 – RUS | -0.33 | 0.05 | -6.10 | **< 0.0001** |
| BpL1 – RUS-RE | -0.21 | 0.05 | -4.35 | **0.0001** |
| L5 – RUS | -0.25 | 0.05 | -5.01 | **< 0.0001** |
| L5 – RUS-RE | -0.12 | 0.04 | -2.86 | **0.022** |
| RUS – RUS-RE | 0.12 | 0.05 | 2.50 | 0.060 |
